# Autumn Royal and Ribier Grape Juice Extracts Reduced Viability and Metastatic Potential of Colon Cancer Cells

**DOI:** 10.1155/2018/2517080

**Published:** 2018-01-14

**Authors:** Manuel Valenzuela, Lorena Bastias, Iván Montenegro, Enrique Werner, Alejandro Madrid, Patricio Godoy, Mario Párraga, Joan Villena

**Affiliations:** ^1^Facultad de Ciencias de la Salud, Universidad Central de Chile, 8330507 Santiago, Chile; ^2^Laboratorio de Comunicaciones Celulares, Centro de Estudios Moleculares de la Célula (CEMC), Program of Cell and Molecular Biology, Institute of Biomedical Sciences (ICBM), School of Medicine, Universidad de Chile, 8380453 Santiago, Chile; ^3^Centro de Investigaciones Biomédicas (CIB), School of Medicine, Universidad de Valparaíso, 2341386 Valparaíso, Chile; ^4^Escuela de Obstetricia y Puericultura, Facultad de Medicina, Campus de la Salud, Universidad de Valparaíso, Angamos 655, Reñaca, 2520000 Viña del Mar, Chile; ^5^Departamento de Ciencias Básicas, Campus Fernando May, Universidad del Biobío, Avda. Andrés Bello, s/n, Casilla 447, 3780000 Chillán, Chile; ^6^Departamento de Química, Facultad de Ciencias Naturales y Exactas, Universidad de Playa Ancha, Avda. Leopoldo Carvallo 270, Playa Ancha, 2340000 Valparaíso, Chile; ^7^Instituto de Microbiología Clínica, Facultad de Medicina, Universidad Austral de Chile, Los Laureles, s/n, Isla Teja, 5090000 Valdivia, Chile

## Abstract

Antioxidants are known to be beneficial to health. This paper evaluates the potential chemopreventive and anticancer properties of phenolic compounds present in grape juice extracts (GJE) from Autumn Royal and Ribier varieties. The effects of these GJE on viability (SRB day assay) and metastatic potential (migration and invasion parameters) of colon cancer cell lines HT-29 and SW-480 were evaluated. The effects of GJE on two matrix metalloproteinase gene expressions (MMP2 and MMP9) were also evaluated via qRT-PCR. In the former, GJE reduced cell viability in both cell lines in a dose-dependent manner. GJE treatment also reduced cell migration and invasion. Moreover, MMP-2 and MMP-9 gene expression diminished depending on extract and on cell type.* Conclusions*. These results provide novel information concerning anticancer properties of selected GJE by revealing selective cytotoxicity and the ability to reduce invasiveness of colon cancer cells.

## 1. Introduction

Epidemiological data and studies carried out in animal models have established that regular dietary intake of fruits and vegetables is protective against cancer [1]. Specifically, grapes (*Vitis vinifera* L.) are attributed to health-promoting effects related to elevated polyphenol contents [2]. The benefits reported include anti-inflammatory, anticarcinogenic, and immune-stimulatory effects, which are thought to be mediated by polyphenol-induced alterations in signaling pathways [3]. The most abundant polyphenols present in grapes include hydroxybenzoic acids, such as gallic acid; hydroxycinnamic acids (caffeic acid); stilbenes (resveratrol); and flavonoids, like anthocyanins, catechins, and others [4, 5]. The biological activities of these compounds have been ascribed to their antioxidant and free radical scavenging properties [6].

Colon cancer is the third most prevalent cancer in adult populations and is responsible for 9% of cancer-related deaths worldwide [7, 8]. However, colon cancer is, to some extent, preventable [9]. For instance, changes in diet and lifestyle have a significant impact on reducing the risk of developing colorectal cancer [10]. Particularly, polyphenols derived from diverse dietary foods or beverages, such as grapes, teas, and turmeric, display chemopreventive and therapeutic properties against colon and other cancer types [11, 12]. In this respect, resveratrol (RSV) (3,5,4′-trihydroxytrans-stilbene), one of the most abundant grape polyphenols, exhibits interesting properties in terms of colon cancer prevention potential. Furthermore, RSV is a recognized antioxidant molecule [13] that reportedly intervenes in different stages of carcinogenesis, for example, initiation, promotion, and progression. This has been reported in numerous experimental models, including cancer cell lines, animal models, and clinical trials [14]. In related* in vitro* and* in vivo* studies, RSV has been shown to possess anticancer potential against several cancer types, including prostate, hepatic, breast, skin, colorectal, and pancreatic cancer [15–18]. Similar observations have been described for anthocyanins and catechins [19]. Importantly, these three phytochemical compounds constitute nearly 50% of grape polyphenols [19]

Tumor metastasis is one the major causes of morbidity in cancer patients [20]. Unfortunately, no therapy has been developed that successfully targets metastasis-associated processes in any human cancer [21]. Metastasis is a process in which cancer cells migrate from primary tumor sites to distant tissues, where secondary tumors are formed [21]. Initially, this requires detachment from the initial tumor, extracellular matrix (ECM) degradation, migration, and intravasation of cancer cells [22]. Importantly, migration and degradation of the ECM appear to represent critical steps during metastasis [22]. For instance, MMP-2 and MMP-9 (also known as type IV collagenases or gelatinases) are capable of degrading most ECM components forming the basal membrane, facilitating tumor cell escape and metastasis [23].

In this study, GJE were obtained from two widely produced grape varieties in Chile (blue grapes Autumn Royal (AR) and Ribier (RB)), and their anticancer properties were analyzed. The investigation considered* in vitro* effects of GJE on viability and metastatic parameters in SW-480 and HT-29 human colorectal carcinoma cell lines. The results show that GJE decreases cell viability in both cell lines in a dose-dependent manner. Importantly, this effect was selective, in that human dermal fibroblasts (HDF) were not affected. Moreover, GJE exposure diminishes metastatic potential of colon cancer cells by reducing migration and invasion of these cells. Additionally, GJE diminishes expression of matrix metalloproteinase genes MMP-2 and MMP-9. The conclusion discusses evidence that AR and RB GJE selectively reduce colon cancer cell proliferation and, more importantly, inhibit the metastatic potential of colon cancer cells.

## 2. Materials and Methods

### 2.1. Plant Material

Blue grapes cv. Autumn Royal and Ribier were harvested from Aconcagua Valley, Valparaíso, Chile, in summer. They were placed in polyethylene bags and transported at 4°C to the laboratory. The stems were manually removed, while damaged and poor quality fruits were eliminated. The samples were packed in polyethylene bags and stored at –32°C until being used.

### 2.2. Grape Juice Extracts (GJE)

Approximately 200 grapes were defrosted, washed with distilled water, and pressed in a stainless steel manual grape crusher at room temperature. The juice was collected and homogenized by manual stirring, kept in polypropylene bottles, sealed, and stored in a cold chamber at –32°C until analysis. Subsequently, GJE were lyophilized, solubilized in 4 mL of distilled water, and filtered (pore size 0.2 *μ*m).

### 2.3. Cell Culture and Grape Juice Extract Treatment

Human colon cancer cell lines SW-480 and HT-29 and human dermal fibroblast (HDF) were maintained in DMEM High glucose medium (Gibco, San Diego, CA, USA), supplemented with 10% (v/v) fetal bovine serum (FBS) at 37°C in a humidified 5% CO_2_ incubator.

For GJE treatment, appropriate volumes of work solutions were added to the medium to reach indicated concentrations (0, 10, 25, 50, or 100 mg/mL) and cells were then incubated for indicated periods of time (24, 48, 72, and 144 hours).

### 2.4. Cell Viability Assay

Cell viability was determined by the Sulforhodamine B (SRB) (Sigma, St Louis, MO, USA) dye assay as previously described [24]. Cells were seeded at density 3 × 10^3^ cells/well in a 96-well plate for 24 h and then treated with GJE as mentioned above. Following treatment, cells were fixed by adding 25 *μ*L of cold 50% (wt/vol) trichloroacetic acid (TCA) followed by incubation for 60 min at 4°C. Plates were washed with deionized water and dried. Then, 50 *μ*L of SRB solution (0.1% wt/vol in 1% acetic acid) was added per well and incubated for 30 min at room temperature. Unbound SRB was removed by washing with 1% acetic acid. Plates were air-dried and protein-bound dye was solubilized with 100 *μ*L of Tris base (10 mM). Optical densities were read in an automated spectrophotometer plate reader at 540 nm.

### 2.5. Cell Migration and Invasion Assays

For cell migration assay, cells were treated with GJE (25 mg/mL) for 24 h and then seeded in a Transwell chamber without Matrigel coating. Cells (2 × 10^5^ cells/well in 300 *μ*L of serum-free medium) were then seeded into the upper chamber, and 0.5 mL medium containing 20% FBS was added to the lower chamber as a chemoattractant. After incubation for 24 h at 37°C in 5% CO2, the upper surface of the porous membrane was wiped with a cotton swab. Cells that migrated to the lower surface of the porous membrane were fixed in 50% methanol and stained with 0.1% crystal violet.

The cell invasion assay was conducted in a similar fashion in a Transwell chamber with Matrigel coating (Sigma) for 48 h. Matrigel was diluted with serum-free medium to a final concentration of 2 mg/mL, and 8 *μ*m-pore polycarbonate membrane filters were coated with 50 *μ*L of Matrigel. In both migration and invasion assays, cell numbers were counted in twenty random fields (×100) per filter, for a total of three filters (*n* = 3).

  % migration = (number of treated cells/number of control cells) × 100
  % invasion = (number of treated cells/number of control cells) × 100

### 2.6. Reverse Transcription-Polymerase Chain Reaction (RT-PCR)

Total RNA was isolated with Trizol reagent, following instructions provided by the manufacturer (Invitrogen, Carlsbad, CA, USA). RNA samples (2 *μ*g) were treated with the RQ1 DNAse (Promega) and reverse-transcribed using the M-MLV reverse transcriptase (Promega, Madison, WI, USA), using the oligo (dT) 15 primers (Promega) as instructed by the manufacturer. MMP-2, MMP-9, and GADPH mRNA expressions were determined by quantitative real-time PCR conducted in a StepOne™ Real-Time PCR System (Applied Biosystem, Foster City, CA, USA). Briefly, each amplification mixture (20 *μ*l) consisted of 10 *μ*l SYBR Green PCR Master Mix 2x (Applied Biosystems), 2 *μ*l primer mix (250 nM each one), 6 *μ*l of nanopure water, and 2 *μ*l of cDNA. Cycling conditions were as follows: 96°C, 10 min; 40 cycles of 96°C, 5 s; 58°C, 30 s; 72°C, 25 s. Reactions were finished with extensions of 72°C for 25 s. Primers used for MMP-2, MMP-9, and GAPDH amplification were as follows:

  MMP-2 (133 bp): 5′-CCTCCCTGCCCCTCCCTTCA-3′ (sense) and 5′-GCTTCTGGCTGGGTCTGTGGC-3′ (antisense)
  MMP-9 M (174 bp): 5′-GCCTTTGGACACGCACGACG-3′ (sense) and 5′-GCCAAAGCAGGACGGGAGCC-3′ (antisense)
  GAPDH (226 bp): 5′-GAAGGTGAAGGTCGGAGTC-3′ (sense) and 5′-GAAGATGGTGATGGGATTTC-3′ (antisense) (see Table 1).

### 2.7. Statistical Analysis

All data are expressed as the means ± SEM taken from at least three independent experiments. Data were processed using INSTAT v3.05 (GraphPad Software, San Diego, CA, USA, https://www.graphpad.com/). Analysis of variance (ANOVA) for multiple comparisons was used as noted. In all cases, *p* < 0.05 was considered significant. All statistical tests were performed with statistical analysis software.

## 3. Results and Discussion

**Figure 1:**
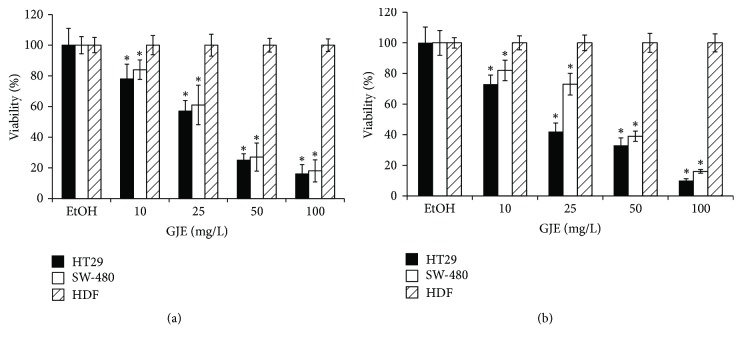
Effect of AR and RB GJE on cell growth of colon cancer cell lines and normal human dermal fibroblast (HDF). Following treatment with the indicated doses of GJE (0, 10, 25, 50, and 100 mg/mL), cell viability was determined by SRB dye assay (see Materials and Methods). Dose-response effects are presented as the percentage of each GJE treatment with respect to EtOH-treated cells (control 100%). The graphs show viability values for HT29 (black bars), SW-480 (white bars), and HDF (slashed bars) in response to AR and RB GJE treatment ((a) and (b), resp.). The data shown are means ± SEM of four independent experiments. ^*∗*^*p* < 0.05 compared with control.

Since* in vitro* cytotoxicity screening models provide important preliminary data to help select grape extracts with potential antineoplastic properties for future study, the cytotoxic effects of the Royal and Ribier grape juice extracts on Human colon cancer cell lines SW-480 and HT-29 were evaluated by the Sulforhodamine B (SRB) assay. The results demonstrate that extracts exhibit significant inhibitory effects in SW-480 and HT-29 at 48 h of treatment (Figure 1). Furthermore, the extracts examined under experimental conditions as described had no cytotoxic effects against normal human dermal fibroblast cells (similar results were obtained with colon epithelial cells (CCD841 CoN), data not shown).

### 3.1. Cell Viability and Cell Migration

#### 3.1.1. Inhibition of Colon Cancer Cell Viability by GJE

To assess effects of GJE treatment on cell viability, SW-480 and HT-29 colon cancer cell lines were exposed to increasing concentrations of AR and RB GJE, and viability was determined using the SRB dye assay (Figure 1). In these dose-response experiments, the lowest GJE dose (10 mg/mL) significantly reduced cell viability in SW-480 and HT-29 cells after 72 h of treatment, as compared to the ethanol control (Figure 1). GJE at a concentration of 25 mg/mL led to significant cell viability reductions in SW-480, 40% and 34% for AR (Figure 1(a)) and RB (Figure 1(b)), respectively, and in HT-29 cells, 43% and 58%, respectively (*p* < 0.001) (Figure 1). The highest GJE concentration (100 mg/mL) induced the most significant reduction in viability for colon cancer cell lines SW-480 (82% and 84% for AR and RB, resp.) and HT29 (84% and 90%, resp.) (*p* < 0.001) (Figures 1(a) and 1(b)). Interestingly, GJE at these same concentrations had no effect on nontumoral cell line derived from human dermal fibroblasts (HDF). These data indicate that AR and RB GJE inhibit the growth of HT-29 and SW-480 colon cancer cells in a dose-dependent manner and that no such detrimental effects are observed in a nontransformed control cell line (HDF).

#### 3.1.2. Inhibition of Colon Cancer Cell Migration by GJE

**Figure 2:**
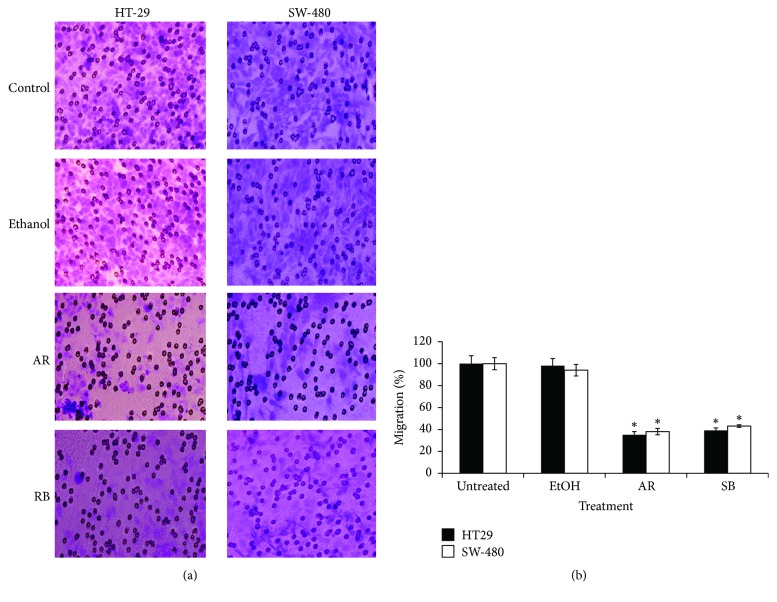
Inhibitory effect of GJE on migration of colon cancer cells. HT-29 and SW-480 cells were treated with AR or RB GJE (25 mg/ml) for 24 h. Following treatment, migration was evaluated in a Transwell chamber (see Materials and Methods). (a) Representative photos of HT-29 and SW-480 migration assays are shown for each indicated condition (magnification ×100). (b) Quantification of the migration assay of SW-480 (white column) and HT-29 (black column). Data represent percentage of migration of treated cells with respect to untreated controls (means ± SEM) from three independent experiments. ^*∗*^*p* < 0.001 versus control.

Given that the GJE under study reduced cell viability of colon cancer cells, research turned to whether GJE might have additional effects on migration. As determined in Transwell assays, AR and RB grape juice extracts significantly reduced migration of HT-29 and SW-480 cells (Figures 2(a) and 2(b)). In Figure 2(a), representative photos of HT-29 migration after treatment with AR and RB GJE (25 mg/mL) are shown. HT-29 cell migration (white bars) was reduced to 35% and 38% of control levels after treatment with AR and RB GJE (25 mg/mL,* time*), respectively. In SW-480 cells, the percentage of migrating cells was reduced to 38% and 44% of control cells following AR and RB GJE treatment (25 mg/ml, 24 h), respectively (Figure 2(b), black columns). This inhibitory effect is significant (*p* < 0.001) in all treatments when compared to controls for both SW-480 and HT-29 cells.

#### 3.1.3. Inhibition of Colon Cancer Cell Invasion by GJE

**Figure 3:**
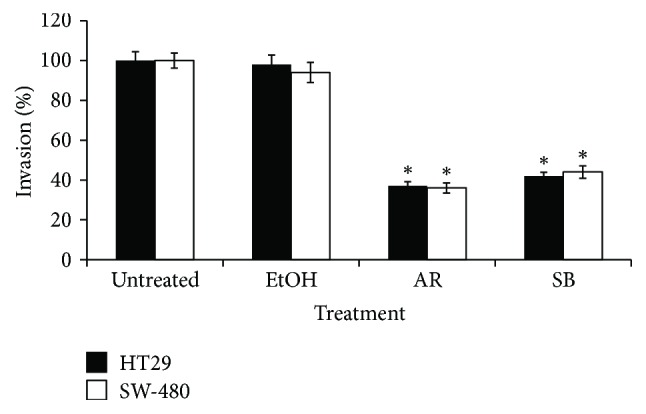
Inhibitory effect of GJE on the invasion capacity of colon cancer cells. HT-29 and SW-480 cells were treated with either AR or RB GJE (25 mg/mL), or EtoH (solvent control) for 48 h, and then harvested and seeded into the upper Transwell chamber coated with Matrigel. Data represent the percentage of the number of cells that invaded the lower chamber with respect to untreated control condition (100%), HT-29 (black column), and SW-480 (white column). Data represent means ± SEM obtained from three independent experiments. ^*∗*^*p* < 0.001 versus. control.

Additionally, the effect of GJE on invasion was determined using a Transwell chamber coated with Matrigel. As is shown in Figure 3, AR and RB GJE significantly reduced invasion of SW-480 and HT-29 cells. In the case of HT-29 cells, AR and RB GJE significantly reduced invasion by 64% and 52%, respectively, when compared to control condition (*p* < 0.001). Similarly, treatment with AR and RB GJE significantly decreased (*p* < 0.001) invasion of SW-480 by 63% and 51%, respectively. In order to elucidate the underlying molecular mechanisms responsible for the observed anti-invasive activity of GJE, changes at the level of MMP-2 and MMP-9 mRNA levels were analyzed via qRT-PCR. As shown in Table 2, AR and RB GJE treatments significantly decreased MMP-2 and MMP-9 mRNA levels in both cell lines. Together, these findings suggest that downregulation of MMP-2 and MMP-9 might be responsible for reduced invasiveness of HT-29 and SW-480 cells observed following GJE treatment.

### 3.2. Discussion

AR and RB GJE display chemoprotective properties against colon cancer, one of the most commonly diagnosed cancers in Western countries. A number of studies have suggested that regular consumption of grapes is associated with the reduced risk of certain cancers, such as breast and colon cancers [25]. The grape juices of Autumn Royal and Ribier exhibited higher phenolics content and health benefits than other species of grape [26]. These extracts are particularly enriched in anthocyanins, with elevated antioxidant capacity compared to other polyphenols present in GJE. Here, we show for the first time that AR and RB GJE reduce viability, migration, and invasive potential of colon cancer cells. These observations are consistent with previous studies where grape seed extracts reduced viability and cell growth in colon cancer cell lines [27, 28]. Interestingly, AR and RB GJE did not modulate HDF cell viability at the same concentrations. Similar results were obtained in studies performed by Zhao et al. [29], who determined that anthocyanin-rich grape extracts were not cytotoxic to normal colon cell line NCM460. Together, these results suggest that this inhibitory effect is restricted to colon cancer cells.

Two important aspects of metastasis are the migratory and invasive ability of tumor cells, which are key characteristics of more aggressive cell phenotypes [30]. Acquisition of these properties allows cancer cells to escape from tissues or organs (affected by primary tumors) to distant sites (producing secondary tumors) [30]. In the present study, a Boyden chamber assay was used to quantify the effect of GJE on migration and invasion parameters of colon cancer cell lines. As expected, GJE inhibited migration in HT-29 and SW-480 colon cancer cell lines. Another study using grape seed proanthocyanidin extracts obtained similar results against lung cancer cell lines A549 and H129. In those cells, effects were attributed to guanylate cyclase and MAP kinase pathway inhibition [30]. Furthermore, GJE also displayed potent inhibitory effects on the invasive potential of colon cancer cells in Matrigel assays. These results are consistent with other studies that found grape seed extracts rich in polyphenols reduced invasion via MMP-2 and MMP-9 expression reduction, for instance, in prostate cancer cell lines [31]. Indeed, the observed effects on invasion and migration in colon cancer cells were correlated here with significant changes in metalloproteinase expression in response to GJE treatment. Metastasis is accompanied by various molecular alterations, including the ability to degrade ECM: this is associated with the overexpression of enzymes displaying proteolytic activity, such as MMPs [32]. Accordingly, augmented expression of MMP-2 and MMP-9 had been shown to play a critical role in degrading basement membranes during cell invasion and migration. Moreover, Bajaj et al. [33] demonstrated that overexpression of MMP-9 is related to tumor invasion and metastasis in gastric carcinoma. Sun et al. [34] reported that the expression of MMP-2 and MMP-9 has significant prognostic value in node-negative patients for predicting relapse-free survival, while Li et al. [35] determined that these MMPs are markers for metastasis in colon cancer. Despite the above observations, to date, the possibility that AR and RB GJE could alter MMP expression in colon cancer cells had not been explored. Indeed, reduced invasive capacity following AR and RB GJE treatment correlated with decreased expression of MMP-2 and MMP-9 genes in both cell lines; however, these results do not exclude the possible participation of other metalloproteinases involved in invasion. For instance, MMP-1 and MMP-7 are also overexpressed in colon cancer [36], and urokinase-PA (u-PA) is implicated in colon cancer metastasis [37]. That said, similar studies on anthocyanins support our results, finding MMP-2 and MMP-9 activity in colon cancer cell line HT-29 [38]. Moreover, a reduction in MMP-2 expression was also observed in prostate cancer cell line DU-145 [30]. Other purified polyphenols present in either grapes or turmeric, that is, myricetin or curcumin, also reduce MMP-2 expression in colon cancer cell lines COLO 205 and HT-29 [39], as well as MMP-9 expression in rat colon cell models [40].

## 4. Conclusions

In summary, we have shown that it is possible to reduce motility-associated processes by exposing colon cancer cells to GJE. These results suggest that the anti-invasive effects of GJE are linked to the inhibition of MMP-mediated degradation processes that favor tumor metastasis. Nevertheless, the underlying molecular mechanisms leading to MMP-2 and MMP-9 inhibition remain to be determined.

## Figures and Tables

**Table 1:** 

Primer	bp	Sense	Antisense
MMP-2	133	5′-CCTCCCTGCCCCTCCCTTCA-3′	5′-GCTTCTGGCTGGGTCTGTGGC-3′
MMP-9	174	5′-GCCTTTGGACACGCACGACG-3′	5′-GCCAAAGCAGGACGGGAGCC-3′
GAPDH	226	5′-GAAGGTGAAGGTCGGAGTC-3′	5′-GAAGATGGTGATGGGATTTC-3′

**Table 2:** Effects of GJE on MMP-2 and MMP-9 gene expression in colon cancer cells. HT-29 and SW-480 colon cancer cells were treated with GJE for 24 h; MMP-2 and MMP-9 mRNA levels were analyzed by quantitative real-time PCR. Results are expressed as the percentage of expression versus control condition. Data represent means ± SEM from three independent experiments. ^*∗*^*p* < 0.001 versus control.

	HT-29		SW-480	
	AR	RB	AR	RB
MMP-2	61.6 ± 1.1^*∗*^	56.8 ± 4.7^*∗*^	90.4 ± 3.9^*∗*^	96.3 ± 2.8^*∗*^
MMP-9	94.9 ± 3.6^*∗*^	97.3 ± 2.4^*∗*^	95.3 ± 4.1^*∗*^	91.3 ± 6.4^*∗*^

## References

[1] Kim H. (2005). New nutrition, proteomics, and how both can enhance studies in cancer prevention and therapy. *Journal of Nutrition*.

[2] Pasinetti G. M., Wang J., Ho L., Zhao W., Dubner L. (2015). Roles of resveratrol and other grape-derived polyphenols in Alzheimer's disease prevention and treatment. *Biochimica et Biophysica Acta (BBA) - Molecular Basis of Disease*.

[3] Xia E.-Q., Deng G.-F., Guo Y.-J., Li H.-B. (2010). Biological activities of polyphenols from grapes. *International Journal of Molecular Sciences*.

[4] Manach C., Scalbert A., Morand C. (2004). Polyphenols, food sources and bioavailability. *The American Journal of Clinical Nutrition*.

[5] Anastasiadi M., Pratsinis H., Kletsas D., Skaltsounis A.-L., Haroutounian S. A. (2010). Bioactive non-coloured polyphenols content of grapes, wines and vinification by-products: Evaluation of the antioxidant activities of their extracts. *Food Research International*.

[6] Russo G. L. (2007). Ins and outs of dietary phytochemicals in cancer chemoprevention. *Biochemical Pharmacology*.

[7] Siegel R. L., Ward E. M., Jemal A. (2012). Trends in colorectal cancer incidence rates in the United States by tumor location and stage, 1992–2008. *Cancer Epidemiology, Biomarkers & Prevention*.

[8] Jemal A., Siegel R., Xu J., Ward E. (2010). Cancer statistics, 2010. *CA: A Cancer Journal for Clinicians*.

[9] Song M., Giovannucci E. (2016). Preventable incidence and mortality of carcinoma associated with lifestyle factors among white adults in the United States. *JAMA Oncology*.

[10] Willett W. C. (1995). Diet, nutrition, and avoidable cancer. *Environmental Health Perspectives*.

[11] Bar-Sela G., Epelbaum R., Schaffer M. (2010). Curcumin as an anti-cancer agent: review of the gap between basic and clinical applications. *Current Medicinal Chemistry*.

[12] Li Y., Kong D., Wang Z., Sarkar F. H. (2010). Regulation of microRNAs by natural agents: an emerging field in chemoprevention and chemotherapy research. *Pharmaceutical Research*.

[13] Olas B., Wachowicz B. (2005). Resveratrol, a phenolic antioxidant with effects on blood platelet functions. *Platelets*.

[14] Patel K. R., Scott E., Brown V. A., Gescher A. J., Steward W. P., Brown K. (2011). Clinical trials of resveratrol. *Annals of the New York Academy of Sciences*.

[15] Benitez D. A., Pozo-Guisado E., Alvarez-Barrientos A., Fernandez-Salguero P. M., Castellón E. A. (2007). Mechanisms involved in resveratrol-induced apoptosis and cell cycle arrest in prostate cancer-derived cell lines. *Journal of Andrology*.

[16] Bishayee A., Dhir N. (2009). Resveratrol-mediated chemoprevention of diethylnitrosamine-initiated hepatocarcinogenesis: inhibition of cell proliferation and induction of apoptosis. *Chemico-Biological Interactions*.

[17] Mo W., Xu X., Xu L. (2012). Resveratrol inhibits proliferation and induces apoptosis through the hedgehog signaling pathway in pancreatic cancer cell. *Pancreatology*.

[18] Sengottuvelan M., Deeptha K., Nalini N. (2009). Resveratrol attenuates 1,2-dimethylhydrazine (DMH) induced glycoconjugate abnormalities during various stages of colon carcinogenesis. *Phytotherapy Research*.

[19] Singh C. K., Siddiqui I. A., El-Abd S., Mukhtar H., Ahmad N. (2016). Combination chemoprevention with grape antioxidants. *Molecular Nutrition & Food Research*.

[20] Partin A. W., Mangold L. A., Lamm D. M., Walsh P. C., Epstein J. I., Pearson J. D. (2001). Contemporary update of prostate cancer staging nomograms (Partin Tables) for the new millennium. *Urology*.

[21] Steeg P. S., Theodorescu D. (2008). Metastasis: A therapeutic target for cancer. *Nature Clinical Practice Oncology*.

[22] Nguyen D. X., Bos P. D., Massagué J. (2009). Metastasis: From dissemination to organ-specific colonization. *Nature Reviews Cancer*.

[23] Itoh Y., Nagase H. (2002). Matrix metalloproteinases in cancer. *Essays in Biochemistry*.

[24] Montenegro I., Tomasoni G., Bosio C. (2014). Study on the cytotoxic activity of drimane sesquiterpenes and nordrimane compounds against cancer cell lines. *Molecules*.

[25] Falcao J. M., Dias J. A., Miranda A. C., Leitao C. N., Lacerda M. M., da Motta L. C. (1994). Red wine consumption and gastric cancer in portugal: A case-control study. *European Journal of Cancer Prevention*.

[26] Lutz M., Jorquera K., Cancino B., Ruby R., Henriquez C. (2011). Phenolics and Antioxidant Capacity of Table Grape (Vitis viniferaL.) Cultivars Grown in Chile. *Journal of Food Science*.

[27] Kaur M., Singh R. P., Gu M., Agarwal R., Agarwal C. (2006). Grape seed extract inhibits in vitro and in vivo growth of human colorectal carcinoma cells. *Clinical Cancer Research*.

[28] Kaur M., Agarwal C., Agarwal R. (2009). Anticancer and cancer chemopreventive potential of grape seed extract and other grape-based products. *Journal of Nutrition*.

[29] Zhao C., Giusti M. M., Malik M., Moyer M. P., Magnuson B. A. (2004). Effects of commercial anthocyanin-rich on colonic cancer and nontumorigenic colonic cell growth. *Journal of Agricultural and Food Chemistry*.

[30] Hanahan D., Weinberg R. A. (2011). Hallmarks of cancer: the next generation. *Cell*.

[31] Engelbrecht A.-M., Mattheyse M., Ellis B. (2007). Proanthocyanidin from grape seeds inactivates the PI3-kinase/PKB pathway and induces apoptosis in a colon cancer cell line. *Cancer Letters*.

[32] Vayalil P. K., Mittal A., Katiyar S. K. (2004). Proanthocycanidins from grape seeds inhibit expression of matrix metalloproteinases in human prostate carcinoma cells, which is associated with the inhibition of activation of MAPK and NF*κ*B. *Carcinogenesis*.

[33] Bajaj G. K., Kleinberg L., Terezakis S. (2005). Current concepts and controversies in the treatment of parenchymal brain metastases: Improved outcomes with aggressive management. *Cancer Investigation*.

[34] Sun W.-H., Sun Y. L., Fang R. N. (2005). Expression of cyclooxygenase-2 and matrix metalloproteinase-9 in gastric carcinoma and its correlation with angiogenesis. *Japanese Journal of Clinical Oncology*.

[35] Li H.-C., Cao D.-C., Liu Y. (2004). Prognostic value of matrix metalloproteinases (MMP-2 and MMP-9) in patients with lymph node-negative breast carcinoma. *Breast Cancer Research and Treatment*.

[36] Okada N., Ishida H., Murata N., Hashimoto D., Seyama Y., Kubota S. (2001). Matrix metalloproteinase-2 and -9 in bile as a marker of liver metastasis in colorectal cancer. *Biochemical and Biophysical Research Communications*.

[37] Kim M., Murakami A., Ohigashi H. (2007). Modifying effects of dietary factors on (-)-epigallocatechin-3-gallate- induced pro-matrix metalloproteinase-7 production in HT-29 human colorectal cancer cells. *Bioscience, Biotechnology, and Biochemistry*.

[38] Lai K.-C., Huang A. N.-C., Hsu S.-C. (2010). Benzyl Lsothiocyanate (BITC) inhibits migration and invasion of human colon cancer HT29 cells by inhibiting matrix metalloproteinase-2/-9 and urokinase plasminogen (uPA) through PKC and MAPK signaling pathway. *Journal of Agricultural and Food Chemistry*.

[39] Yun J. W., Lee W. S., Kim M. J. (2010). Characterization of a profile of the anthocyanins isolated from Vitis coignetiae Pulliat and their anti-invasive activity on HT-29 human colon cancer cells. *Food and Chemical Toxicology*.

[40] Ko C. H., Shen S. C., Lee T. J. (2005). Myricetin inhibits matrix metalloproteinase 2 protein expression and enzyme activity in colorectal carcinoma cells. *Molecular Cancer Therapeutics*.

